# Enhanced electrocatalytic hydrogen generation from water *via* cobalt-doped Cu_2_ZnSnS_4_ nanoparticles†

**DOI:** 10.1039/c8ra01886c

**Published:** 2018-06-04

**Authors:** Renuka V. Digraskar, Vijay S. Sapner, Shankar S. Narwade, Shivsharan M. Mali, Anil V. Ghule, Bhaskar R. Sathe

**Affiliations:** Department of Chemistry, Dr Babasaheb Ambedkar Marathwada University Aurangabad 431004 Maharashtra India bhaskarsathe@gmail.com; Department of Chemistry, Shivaji University Kolhapur Maharashtra India

## Abstract

Herein, we adopted a novel noble metal-free Co-doped CZTS-based electrocatalyst for the hydrogen evolution reaction (HER), which was fabricated using a facile, effective, and scalable strategy by employing a sonochemical method. The optimized Co-doped CZTS electrocatalyst shows a superior HER performance with a small overpotential of 200 and 298 mV at 2 and 10 mA^−1^, respectively, and Tafel slope of 73 mV dec^−1^, and also exhibits excellent stability up to 700 cycles with negligible loss of the cathodic current. The ease of synthesis and high activity of the Co-doped CZTS-based cost-effective catalytic system appear to be promising for HER catalysis.

## Abstract 2

A novel noble metal-free Co-doped CZTS-based nano-electrocatalyst fabricated by employing a sonochemical method for the enhanced hydrogen evolution reaction (HER) and it shows a superior HER performance and exhibits excellent current stability.

## Graphical abstract

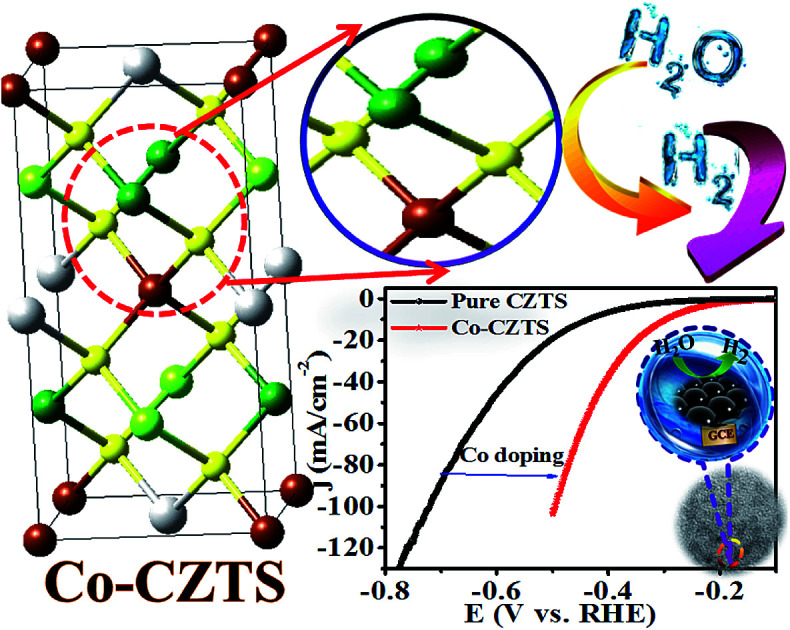

## 1. Introduction

Intensification of the conflict caused by energy security with declining natural energy reserves and the increasing environmental problems together with the urge for the development of modern society have created a huge demand for futuristic renewable and environmentally friendly energy sources. Hydrogen (H_2_), as an ideal clean energy carrier, is proposed to be a major energy resource for the future world.^1^ Currently, the traditional methods for the large-scale production of H_2_ involve the release of a large amount of CO_2_, high energy consumption and multistep and costly processes such as natural gas reforming and gasification of coal and petroleum coke.^2^ Thus, considerable efforts have been directed towards the sustainable production of H_2_ from water electrolysis as an emerging clean-energy technology.^3^ Therefore, efficient cathode materials are needed to reduce the overpotential and improve the efficiency of the hydrogen evolution reaction (HER). Currently, expensive platinum (Pt) and Pt-based electrocatalysts are the most effective and electrochemically stable catalysts commonly used in the HER.^4^ Unfortunately, their scarcity and high cost limit their utilization on a large-scale for commercial applications. Thus, it is crucial and a key challenge to develop earth-abundant and inexpensive noble metal-free catalysts that possess good activity with high stability for the HER. Recently, many potential alternatives such as transition-metal dichalcogenides,^5,6^ carbides,^7,8^ phosphides,^9^ boride^10^ and nitrides^11^ have been reported as efficient electrocatalysts for the HER. Among them, transition-metal dichalcogenides (TMDs), *i.e.* MoS_2_, CoS_2_, and WS_2_, have attracted special attention due to their earth-abundant nature and promising properties as HER electrocatalysts. However, one of the challenges with layered dichalcogenide electrocatalysts is their poor electrical conductivity and small number of active sites, which are limited only to the edges, although some are reported to convert their thermodynamically favoured semiconducting phase to a metastable metallic polymorph.^9,12–14^ Thus, Tan *et al.* reported the non-layered metal dichalcogenide CoS_2_ having an evident advantage over the layered metal dichalcogenides due to its conducting nature.^9^ In our previous work, we revealed that CZTS electrocatalysts show excellent HER activity, and thus more efforts have been made to further improve their HER activity. Interestingly, doping is one of the best strategies to further improve the catalytic performance. Several doped electrocatalysts have been reported, for instance, Liang *et al.* demonstrated an Fe-promoted MoP catalyst for enhanced HER performance compared to MoP.^15^ Liu *et al.* reported that an FeP/NCNT hybrid exhibited higher HER activity and stability than FeP NPs.^16^ Gao *et al.* reported N-doped WS_2_ nanosheets, which exhibit high electrocatalytic activity.^17^ Wang *et al.* demonstrated that an Ni-doped MoS_2_ catalyst shows excellent HER activity compared to pure MoS_2_.^18^ More interestingly, the current research in this area is focused on using transition metals (Fe, Co, Ni, Cu, Cr, and Zn) doped with boride,^10^ phosphide,^19^ carbide,^20^ and chalcogenide (S and Se), such as MoS_2_, WS_2_, and FeS_2_,^21–24^ which have been found to be highly efficient catalysts for the HER. However, their preparation requires a gas chamber, costly and harmful chemicals, and very careful handling, thus prompting researchers to find cheaper alternatives. Nevertheless, to date, reports on the doping effect on the catalytic performance of Co-CZTS hydrogen evolution hybrid catalysts are rare. Based on the above research progress and corresponding HER studies, herein, we investigate improving the performance of the HER with the chalcogenide Cu_2_ZnSnS_4_ (CZTS) successfully doped with Co, which has a low cost, high earth abundance, small size, high current density and stability even in harsh acidic conditions and is promising for the HER by water splitting.

## 2. Experimental section

### 2.1 Chemicals

Copper chloride (CuCl_2_·2H_2_O, 98%), zinc chloride (ZnCl_2_·2H_2_O, 96%), tin chloride (SnCl_2_·2H_2_O, 98%), thioacetamide (TAA), cobalt chloride (CoCl_2_·6H_2_O), 2-methoxyethanol, monoethanolamine (MEA) and absolute ethanol of AR grade were used for the sonochemical synthesis of Co-doped CZTS nanoparticles. All the chemicals were procured from Sigma Aldrich and were used as received without any further purification.

### 2.2 Synthesis of Co-doped CZTS nanoparticles

Initially, the sol was prepared with the Cu : Zn : Sn : TAA ratio of 2 : 1 : 1 : 4 using the respective salt solutions. 1 M of CuCl_2_ was dissolved in 100 mL of 2-methoxyethanol under vigorous stirring for 30 min followed by the addition of 0.5 M ZnCl_2_ and stirring for 15 min. Then, 0.5 M SnCl_4_ was added with constant stirring for 15 min to obtain a clear solution. To this solution, 2 M TAA and an appropriate amount of monoethanolamine were added under continuous and vigorous stirring until the solution turned dark brown. Further, controlled doping was carried by the addition of 5 wt% CoCl_2_ (doping amount) to this solution, which was then subjected to ultrasonication in an ultrasonic bath (40 kHz, 40 W) for 1.5 h to achieve a black precipitate. The precipitate was repeatedly washed using absolute ethanol to remove excess TAA and other counter ions and then annealed at 170 °C (decomposition temperature of metal–TAA complex) for 2 h.^25^ This as-synthesized Co-doped CZTS nanoparticles were further characterized and tested as an electrocatalyst for the HER. Interestingly, the electrocatalytic studies towards the water splitting reactions of the sonochemically synthesized CZTS nanoparticles are considered a point of interest in this work.

### 2.3 Structural and morphological characterizations

The product was analyzed *via* Fourier transform infrared spectroscopy (FTIR). Its phase and structure were characterized using X-ray diffraction (XRD, Siemens D-5005 diffractometer) equipped with an X-ray tube (Cu Kα; *λ* = 1.5418 nm, 40 kV, 30 mA, with a step size of 0.01°). X-ray photoelectron spectroscopy (XPS) on a SPECS HSA-3500 with a monochromatic Al K_α_ X-ray radiation X-ray source and hemispherical analyser was used to investigate the elemental states of the sample. The BET surface area of the CZTS powder-based catalyst was determined *via* N_2_ adsorption at 77 K and isotherm measurements at 77.3 K using a Quantachrome NovaWin© 1994–2012, Quantachrome Instruments v11.02. Raman spectroscopy was performed using a Raman optical microscope, Seki Technotron Corp. Tokyo with a 532 nm laser. The electrochemical and electrocatalytic studies were performed using an electrochemical workstation (CHI-Instrument 660E, USA) with a three-electrode system.

### 2.4 Electrochemical measurements

The electrochemical HER activity of the catalysts was tested by cyclic voltammetry (CV), linear sweep voltammetry (LSV) and electrochemical impedance spectroscopic (EIS) analysis. The measurements were performed on an electrochemical workstation (CHI-660E) using a three-electrode system with a modified glassy carbon electrode (GCE; 3 mm in dia.) as the working electrode, and saturated calomel electrode (SCE) and platinum wire as the reference and counter electrodes, respectively. Prior to use, the GCE was polished with 1 μm, 0.3 μm and 0.05 μm alumina powder, respectively, followed by sonication in deionized water and methanol for 5 min each. For the fabrication of the working electrode, the catalyst ink was prepared by dispersing 5.0 mg of catalyst into a mixed solvent containing 100 : 1 of isopropanol : Nafion (5 wt%) solution and sonicating the mixture for ∼30 min to form a homogeneous ink. Afterwards, 10 μL (0.41 mg loading normalized to current density) of the catalytic ink was loaded onto the GCE and dried naturally at RT. Cyclic voltammetry (CV) and linear sweep voltammetry (LSV) were conducted in 0.5 M aqueous H_2_SO_4_ electrolytic solutions. All the results are reported with respect to the reversible hydrogen electrode (RHE) in 0.5 M H_2_SO_4_, where *E* (RHE) = *E* (SCE) + 0.244 V. Electrochemical impedance spectroscopy (EIS) measurements were carried out from 1 000 000 Hz to 0.002 Hz at a slightly higher onset potential (−0.21 V *vs.* RHE).

## 3. Results and discussion

**Scheme 1:**
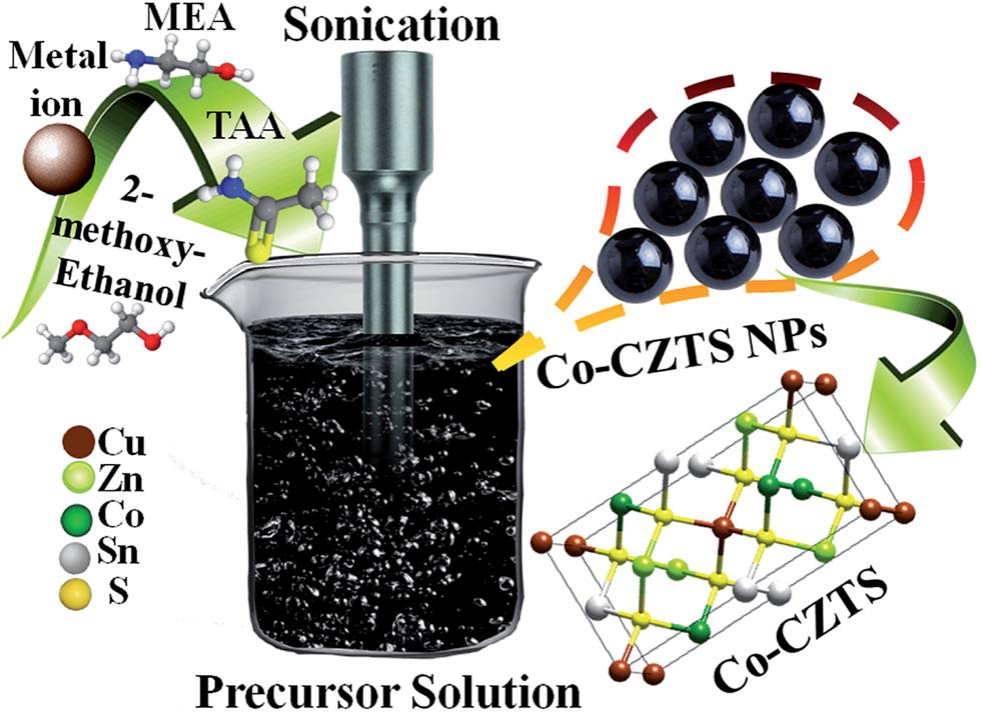
Schematic illustration of the synthetic process for the Co-doped CZTS NPs.

**Fig. 1:**
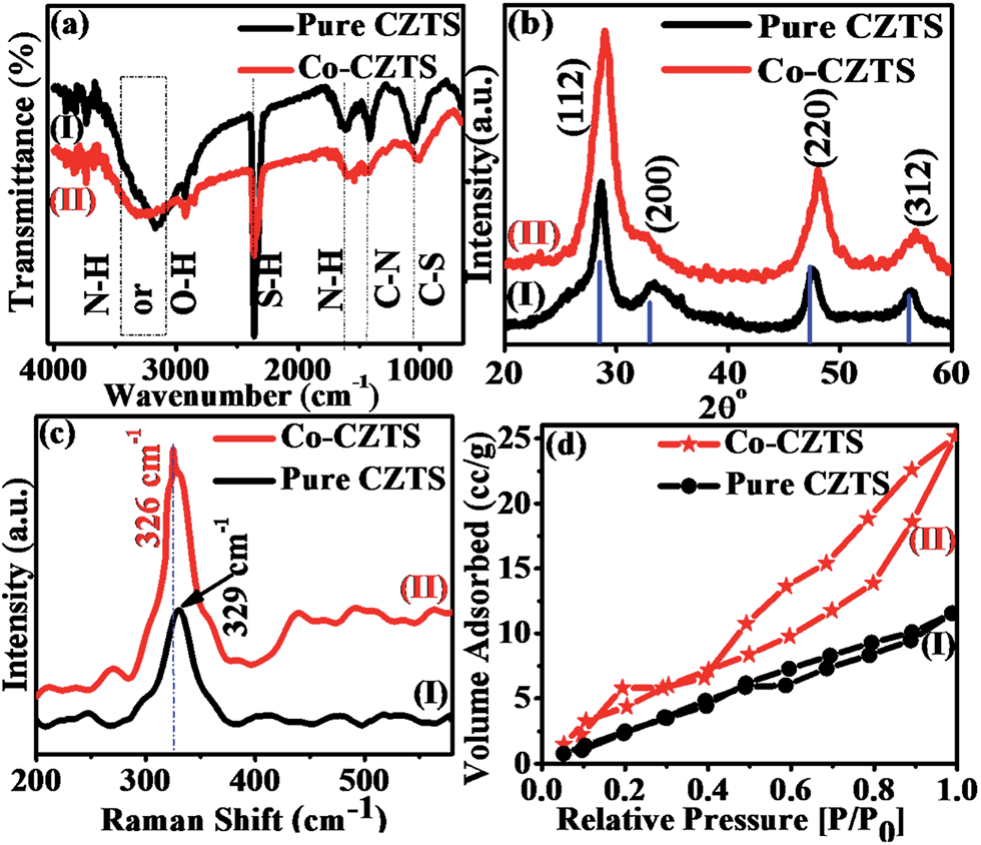
Superimposed (a) FTIR, (b) XRD and (c) Raman spectra and (d) BET curves of CZTS (I) and Co-doped CZTS (II).

Scheme 1 shows a schematic of the synthesis of the Co-doped CZTS nanoparticles. FT-IR spectroscopy is usually employed to probe the various functional groups and inorganic and organic species and their inter- and intra-binding in samples. Thus, the undoped and Co-doped CZTS nanostructured samples were characterized *via* FT-IR spectroscopy for comparison in the spectral range of 400–4000 cm^−1^, as shown in Fig. 1(a). The spectra of the undoped and Co-doped CZTS samples show a common broad band at 3440 cm^−1^, which is attributed to the O–H stretching vibration,^26^ while the band at 1020 cm^−1^ corresponds to the C–S stretching. The bands at 1430 and 1650 cm^−1^ are due to the coupled vibrations of the C–N stretching and N–H bending in the precursor complex, respectively.^27^ The band at 2360 cm^−1^ is attributed to the S–H thiol functionality, which is in agreement with the literature value.^28^ Moreover, there are no obvious differences between the doped and undoped samples, suggesting that no new bonds are formed in the doped sample in the IR frequency range.

Fig. 1(b) shows the representative superimposed powder XRD patterns of the CZTS (I) and Co-doped CZTS (II) nanoparticles. The diffraction peaks in the spectra of both samples are indexed to the kesterite structure of CZTS, and the lattice parameters were calculated to *a* = 5.399 Å and *c* = 10.873 Å for pure CZTS and *a* = 5.396 Å and *c* = 10.871 Å for the Co-doped CZTS. These values are consistent with the literature values (*a* = 5.427 Å and *c* = 10.848 Å, JCPDS no. 26-0575), thus the samples belong to the *I*42*m* space group.^29,30^ No extraneous peaks are observed, indicating the high purity of the final product. The average crystallite size obtained for the pure CZTS and Co-doped CZTS nanoparticles using the Scherrer equation is 4.0 and 3.2 nm and W–H plot of 5.2 and 4.6 nm, respectively. Significantly, upon doping with Co, a slight contraction in the lattice is observed, resulting in broadening of the diffraction peaks, which is in agreement with the literature.^31^ Moreover, the Raman and X-ray photoelectron spectroscopy (XPS) measurements give further evidence for the incorporation of Co into the CZTS lattice. Raman spectroscopy is a useful tool for the analysis of the effects of doping on nanomaterials because the incorporation of dopants leads to shifts in the lattice Raman vibrational peak positions. Accordingly, as shown in Fig. 1(c), the Raman spectra of the CZTS (I) and Co-doped (II) CZTS nanoparticles exhibit a strong peak located at 329 cm^−1^, which is indirect evidence of the formation of CZTS NPs. Moreover, after the doping of Co in CZTS, this peak shifts towards a lower energy of 326 cm^−1^, which can be attributed to electron molecular vibrational coupling due to the substitution of Co^2+^ ions in the host lattice or (Zn^2+^ ions).^32^ Accordingly, the increased intensity of this mode for the cobalt-doped sample compared to the undoped sample suggests that the Co-doped sample contains a higher density of defects and lattice disorders, which is in agreement with literature.^33^ It is also important to note that there is no additional peak in the Raman spectra of the CZTS and Co-doped CZTS samples, which further confirms the absence of any spurious (and/or impurity) phases of dopant in the samples according to the literature survey.^32^ Fig. S1, ESI† presents the X-ray photoelectron spectra (XPS) of the samples, which further support our earlier findings of the incorporation of Co into the host lattice of the Cu_2_ZnSnS_4_ nanocrystals, where the cobalt atoms are Co^2+^ substituting the Zn^2+^ lattice sites in CZTS. The lower binding energy of the 2p^3/2^ peak at 778.1 eV by 15.6 eV is attributed to cobalt metal (Co^0^),^34^ and the detailed elucidation is presented in the ESI.† As shown in Fig. 1(d), the BET surface area of the Co-doped CZTS is 4.213 m^2^ g^−1^, which is slightly higher than the 2.016 m^2^ g^−1^ of CZTS due to the smaller particle size, pore-size distribution and pore volume of the undoped sample. The CZTS and Co-doped CZTS nanoparticles exhibited an average pore size of 3.578 and 4.617 nm and pore volume of 0.017 and 0.77 cm^3^ g^−1^, respectively. The higher surface area of the doped CZTS suggests it has enhanced electrocatalytic activity.

**Fig. 2:**
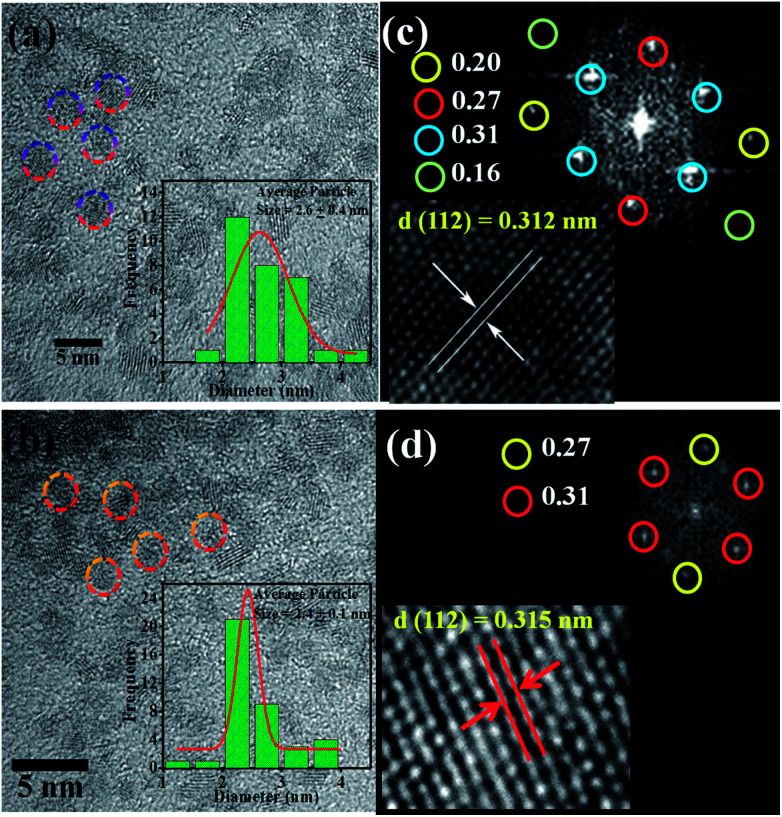
HRTEM image of (a) pure CZTS NPs (2.6 ± 0.1 nm) and (b) Co-doped CZTS NPs (2.4 ± 0.1 nm) (inset shows the particle size distribution plots). Fast-Fourier transform (FFT) patterns of (c) pure CZTS NPs and (d) Co-doped CZTS NPs (inset shows the lattice fringes).

Fig. 2 shows the morphology of the CZTS nanoparticles before and after Co doping. Fig. 2(a) shows that the undoped CZTS nanoparticles are spherical with a uniform particle size distribution. The circled crystalline particles in Fig. 2(b) show the presence of several small-sized well dispersed CZTS NPs without any agglomeration with a slight difference in population. From the images it is clear that the morphology of CZTS did not change after Co doping. Moreover, (inset in Fig. 2(a) and (b)) the particle size distribution histograms of the pure and Co-doped CZTS nanoparticles, respectively, demonstrate that their average particle size decreased because Co substituted Zn in the lattice sites, which creates defects.^35^ The particle size distribution was 2.6 ± 0.4 and 2.4 ± 0.1 for the pure CZTS and Co-doped CZTS, respectively, which is relatively lower than that reported for samples synthesized *via* other methods in the literature.^36,37^ Significantly, the reduction in particle size after Co doping in CZTS, where Co substituted Zn in the lattice sites, is attributed to the smaller ionic radius of Co^2+^ (0.72 Å) than that of Zn^2+^ (0.74 Å), which is in good agreement with the XRD results. Consequently, the substitution of Zn by Co causes a decrease in the lattice constant.^32^ Accordingly, the fast-Fourier transform (FFT) pattern of the Co-doped CZTS nanoparticles in Fig. 2(c) corresponds to the diffraction spot reflecting the *d*-spacings of 3.1 and 2.7 Å, which can be indexed to the 112 and 200 planes, respectively. This is in agreement with the XRD analysis shown in Fig. 1(b). Moreover, lattice fringes with the *d*-spacing of 0.312 and 0.315 nm are also observed for the pure CZTS and Co-doped CZTS, which correspond to the (112) representative plane of CZTS, as shown in Fig. 2(c) and (d), respectively.

### 3.1 Electrochemical studies

**Fig. 3:**
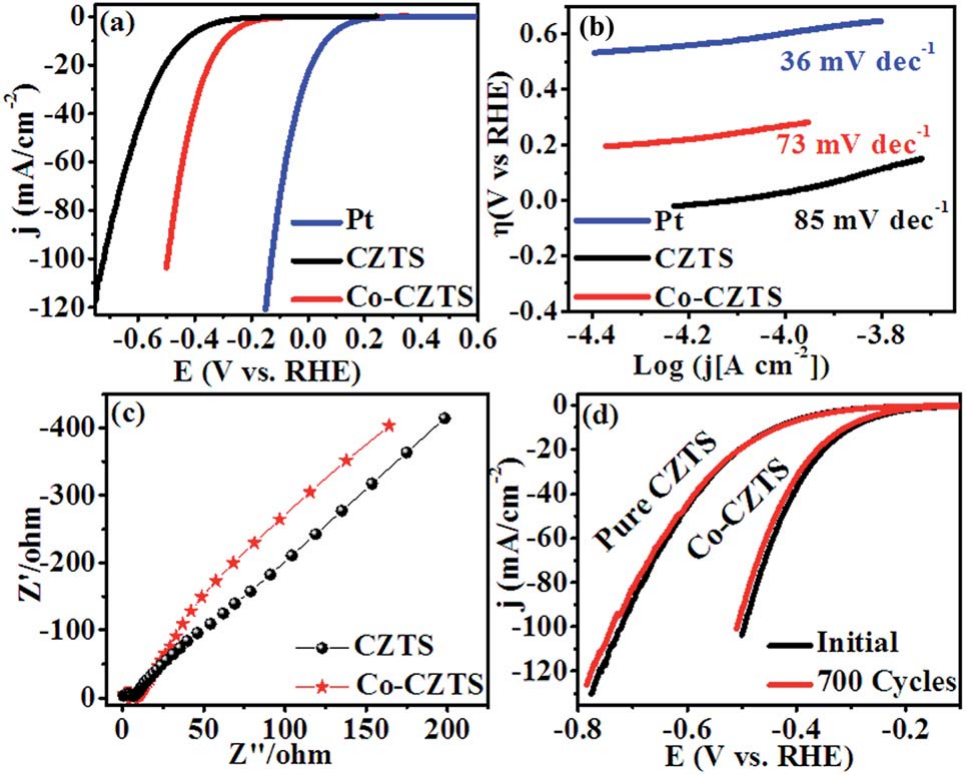
(a) Polarization curves, (b) corresponding Tafel plots and (c) Nyquist plots of the CZTS and Co-doped CZTS NPs. (d) Durability test of Co-CZTS in 0.5 M H_2_SO_4_.

To demonstrate the enhancement in electrocatalytic activity CZTS after Co doping, cyclic voltammetry (CV) and linear sweep voltammetry (LSV) measurements were carried out and the results compared with that obtained for a Pt catalyst at a scan rate of 50 mV s^−1^*vs.* RHE in 0.5 M H_2_SO_4_ solution. As expected, Pt exhibited a much higher HER catalytic performance than the as-synthesised CZTS electrocatalysts.^38^ After doping Co (low-cost and earth-abundant element) in CZTS, its catalytic activity towards the HER was significantly promoted. Accordingly, Fig. 3(a) presents the superimposed LSV curves of the reference Pt, CZTS and Co-doped CZTS, which shows that the electrocatalytic performance of Co-CZTS is better than that of CZTS with an overpotential of 200 and 298 mV at 2 and 10 mA^−1^, respectively, and it exhibits a higher current density of −103 mA cm^−1−2^ (see Scheme S1 in ESI†). A slight change in the features of the LSV curves at a scan rate from 10–100 mV s^−1^*vs.* RHE is observed, which reveals that the catalytic activity of the Co-doped and undoped CZTS NPs toward the HER could be due to controlled mass transfer processes at the electrocatalytic interfaces (see S2 ESI†). Table 1 shows a comparison of the HER performance of the catalysts in the present work with that of other electrocatalysts reported in the literature. From the table, we observe that the low overpotential and Tafel slope of CZTS indicate that it is a promising material for the HER. Since the Tafel slope represents the intrinsic activity of HER catalysts, a smaller Tafel slope value represents a faster HER rate with respect to the increase in overpotential (positive shift). The Tafel slopes derived from the polarization curves were fitted into the Tafel equation (*η* = *a* + *b* log *j*, where *b* is the Tafel slope and *j* is the current density). As shown in Fig. 3(b), the commercial Pt/C catalyst still shows the smallest Tafel slope of ∼36 mV dec^−1^, which is close to the reported values.^39,40^ Remarkably, the Co-doped CZTS catalyst exhibits a smaller Tafel slope of 73 mV dec^−1^ than that of the pure CZTS catalyst (85 mV dec^−1^), demonstrating its improved HER activity.^41–43^ Also, the exchange current density of the pure and Co-doped CZTS was 882 and 989 mA cm^−2^, respectively.

**Table 1:** Comparison of the electrocatalytic HER performance of non-noble metal electrocatalysts with noble metal representative *i.e.* Pd/C and Co-CZTS (this work) in 0.5 M H_2_SO_4_

Sr. no.	Materials	Overpotential (mV *vs.* RHE)	Tafel slope (mV dec^−1^)	Ref.
1	F–MoS_2_	380	175	46
2	Co@C	284	129.1	47
3	Bulk WS_2_	290	119	48
4	ZFO-700	377	178	49
5	Pd/C	264	124	50
6	MWCNTs@Cu	366	109	51
7	MoP_2_/Mo	273	69	52
8	CoTe NTs	345.4	58.7	53
9	HCL-Ni@C	440	194	54
10	CoP	383	90	55
11	Cu/rGO	−430	207	56
12	MoS_2_ NS	280	90	40
13	FeP NP	292	86	16
14	Ni_12_P_5_	380	270	57
15	N,P-graphene-1	420	145	58
16	Mo_2_C@NPC	260	126.4	8
17	Co_9_S_8_	224	135	59
18	Pristine MoS_2_	200	91	60
19	CZTS	300	85	25
20	Co-CZTS	200	73	This work

The significant enhancement in the final HER activity is mainly attributed to the synergistic effect of Co-doping in the CZTS NPs, which further enriched their defect structure and activated some unreactive edge sites.^23^ We further investigated the HER kinetics of the pure CZTS and Co-doped CZTS catalysts at the electrode/electrolyte interface *via* electrochemical impedance spectroscopy (EIS). The charge-transfer resistance (*R*_ct_) is related to the electrocatalytic kinetics, where a lower value reflects a faster reaction rate, which can be obtained from the diameter of the semicircle in the EIS-Nyquist plot in the low frequency region. Accordingly, as shown in Fig. 3(c), the charge transfer resistance (*R*_ct_) of CZTS (6 Ω) is higher than that of Co-CZTS (4 Ω), which indicates its poor conductivity. Thus, the doping of Co significantly improves the electron transport ability of CZTS during the HER process, suggesting that the incorporation of Co increases its electrical conductivity.^23,44^ Another important criterion for a good electrocatalyst is its current stability for a long period. To assess this, a continuous long-term operation of 700 HER cycles at a 50 mV s^−1^ scan rate was conducted in 0.5 M H_2_SO_4_ for the undoped and Co-doped CZTS catalyst. As shown in Fig. 3(d), a slight loss in activity was observed after 700 cycles, indicating the good durability of the catalysts in acidic media, which is consistent with that reported for CZTS-based systems.^45^ Furthermore, the turnover frequency (TOF) values were estimated for the HER in a 0.5 M H_2_SO_4_ solution using the method of Eric J. Popczun (see ESI† for detailed calculation) at *η* = 300 mV. The TOF of the Co-doped CZTS was calculated to be about 0.188 s^−1^, which is much larger than that of pure CZTS and indicates its superior intrinsic HER catalytic activity. Further, to highlight the significance of this report, our results were compared with that obtained in previous reports. The results demonstrate that the Co-doped CZTS electrode exhibits excellent electrocatalytic activity toward the HER (TOF comparison with other electrocatalysts see ESI Table S1†).

## 4. Conclusion

In summary, a facile sonochemical synthetic strategy has been developed to prepare Co-doped CZTS. XRD, Raman spectroscopy, HR-TEM and XPS were used to achieve a better understanding of the phenomena that occur when Co is incorporated into CZTS. The higher surface area of the Co-doped CZTS indicates its enhanced electrocatalytic activity. With the addition of Co, the HER activity of CZTS was greatly enhanced with a lower overpotential of 163 mV, smaller Tafel slope of 73 mV dec^−1^, high stability even after 700 cycles and smaller charge transfer resistance. We believe that this work not only provides a low-cost and highly active HER system, but also open a new avenue for exploring the use of metal doping in chalcogenides as attractive catalyst materials for electrocatalytic applications in energy conversion devices.

## Conflicts of interest

There are no conflicts to declare.

## Supplementary Material

RA-008-C8RA01886C-s001
