# Supplementary material for: Enhanced electrocatalytic hydrogen generation from water via cobalt-doped Cu2ZnSnS4 nanoparticles
Source: RSC Adv. 2018 Jun 4;8(36):20341–6. doi: 10.1039/c8ra01886c (PMC9080824; doi:10.1039/c8ra01886c)
Supplement: RA-008-C8RA01886C-s001 [file RA-008-C8RA01886C-s001.pdf]

Electronic Supplementary Material (ESI) for RSC Advances.
This journal is © The Royal Society of Chemistry 2018

Electronic Supplementary Material (ESI)

Enhanced Electrocatalytic Hydrogen Generation from Water by Cobalt doped Cu_2ZnSnS_4 Nanoparticles

Renuka V. Digraskar,[a] Vijay S. Sapner[a], Shankar S. Narwade,[a] Shivsharan M. Mali,[a] Anil V. Ghule,[b] Bhaskar R. Sathe[a]*

[a]*Department of Chemistry, Dr. Babasaheb Ambedkar Marathwada University, Aurangabad 431004, Maharashtra, India*

[b]*Department of Chemistry, Shivaji University, Kolhapur 416004,Maharashtra, India*

*Corresponding author: Dr. Bhaskar R. Sathe (bhaskarsathe@gmail.com)

Telephone: +91-8275306471

X-ray photoelectron spectroscopy:

Figure S1 represents the X-ray photoelectron spectroscopic (XPS) measurements to further support our earlier findings of the incorporation of Co- doping into the host lattice of Cu_2ZnSnS_4 nanocrystals. Accordingly, superimposed high-resolution XPS analysis of the pure CZTS nanoparticles shows the oxidation states of constituents element. The peak of Cu 2p split into 932. 6 ($2p_{3/2}$) and 952.5 eV ($2p_{1/2}$) with a splitting energy of 19.9 eV was assigned to the binding energy (BE) of Cu (I). The peak of Zn 2p appeared at BE's of 1022.1 ($2p_{3/2}$) and 1045.1 eV ($2p_{3/2}$) with a splitting energy of ~23.0 eV, which was consistent with the value of Zn (II). The peak of Sn 3d split into 486.6 ($2p_{5/2}$) and 495.1 eV ($2p_{3/2}$) with a splitting energy of 8.5 eV was attributed to Sn (IV). The peak of S 2p exhibited at 162.2 ($2p_{3/2}$) and 163.3 eV ($2p_{1/2}$) with an energy difference of 1.1 eV was consistent with the S range between 160 and 164 eV in the representative sulfide phase. [1, 2]

To confirm the oxidation states of the metals and sulfur in the Co-doped CZTS nanoparticles, we employed XPS, as shown in Figure S1. Cu $2p_{1/2}$ and $2p_{3/2}$ peaks at 952.9 and 932.2 eV are indicative of Cu (I) with a splitting of 20.7 eV. The peak of Zn 2p appeared at BE's of 1021.2 ($2p_{3/2}$) and 1044.4 eV ($2p_{3/2}$) with a splitting energy of 23.2 eV, which was consistent with the value of Zn (II) which is slightly shift towards lower energy after Co doping (which can be attributed to the Co^{2+} ions incorporation in to the Zn^{2+} lattice sites), Co $2p_{1/2}$ and $2p_{3/2}$ peaks at 793.7 and 778.1 eV are indicative of Co (II) with a splitting of 15.6 eV. [3] Sn $3d_{3/2}$ and $3d_{5/2}$ peaks for Sn (IV), at 495.5 and 487.0 eV, show a splitting of 8.5 eV. The peak at 163.6 eV is consistent with literature reports of the S 2p peak in sulfide phases. [3, 4] As can be seen from the spectra, no other valences such as Cu (II) and Sn (II) were detected, and it is not likely that secondary phases CuS, ZnS, SnS, CoS and Cu_2SnS_3 coexist.

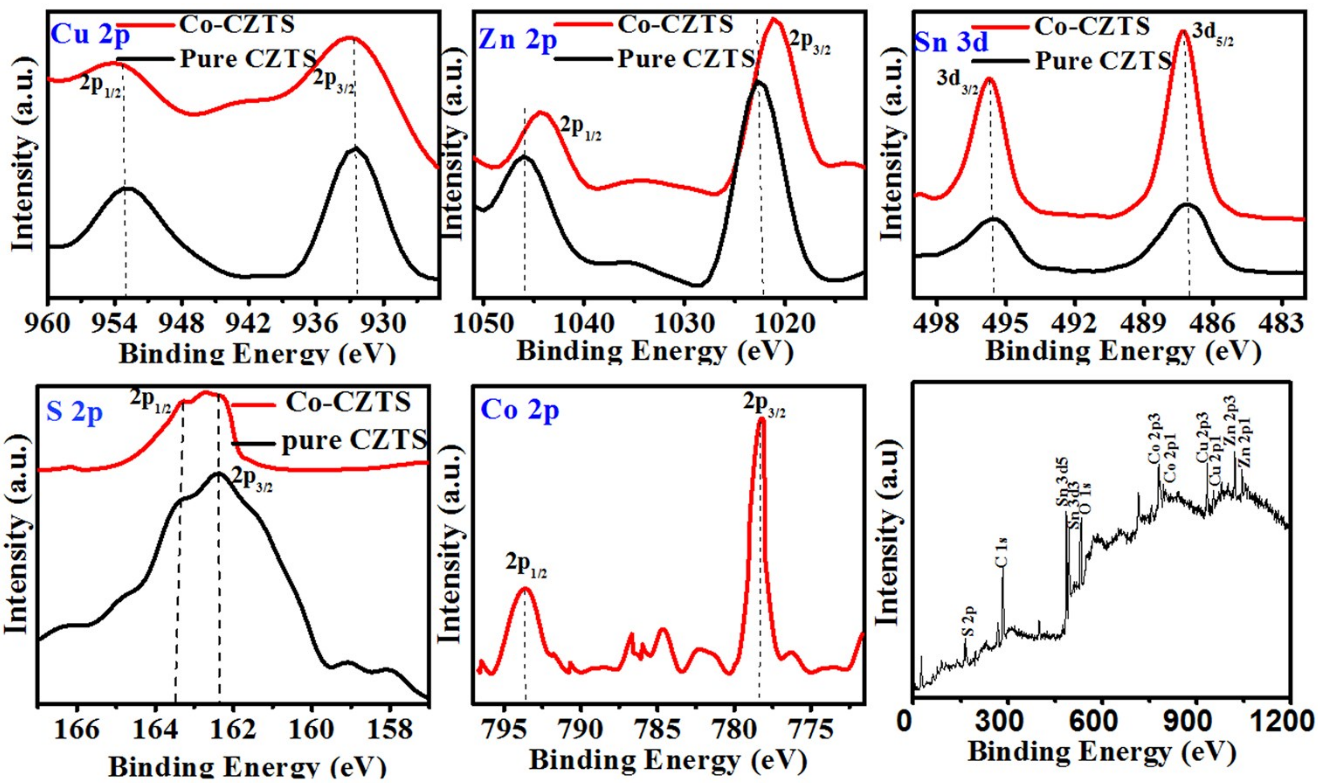

Figure S1: XPS spectra for the CZTS and Co-doped CZTS nanoparticles.

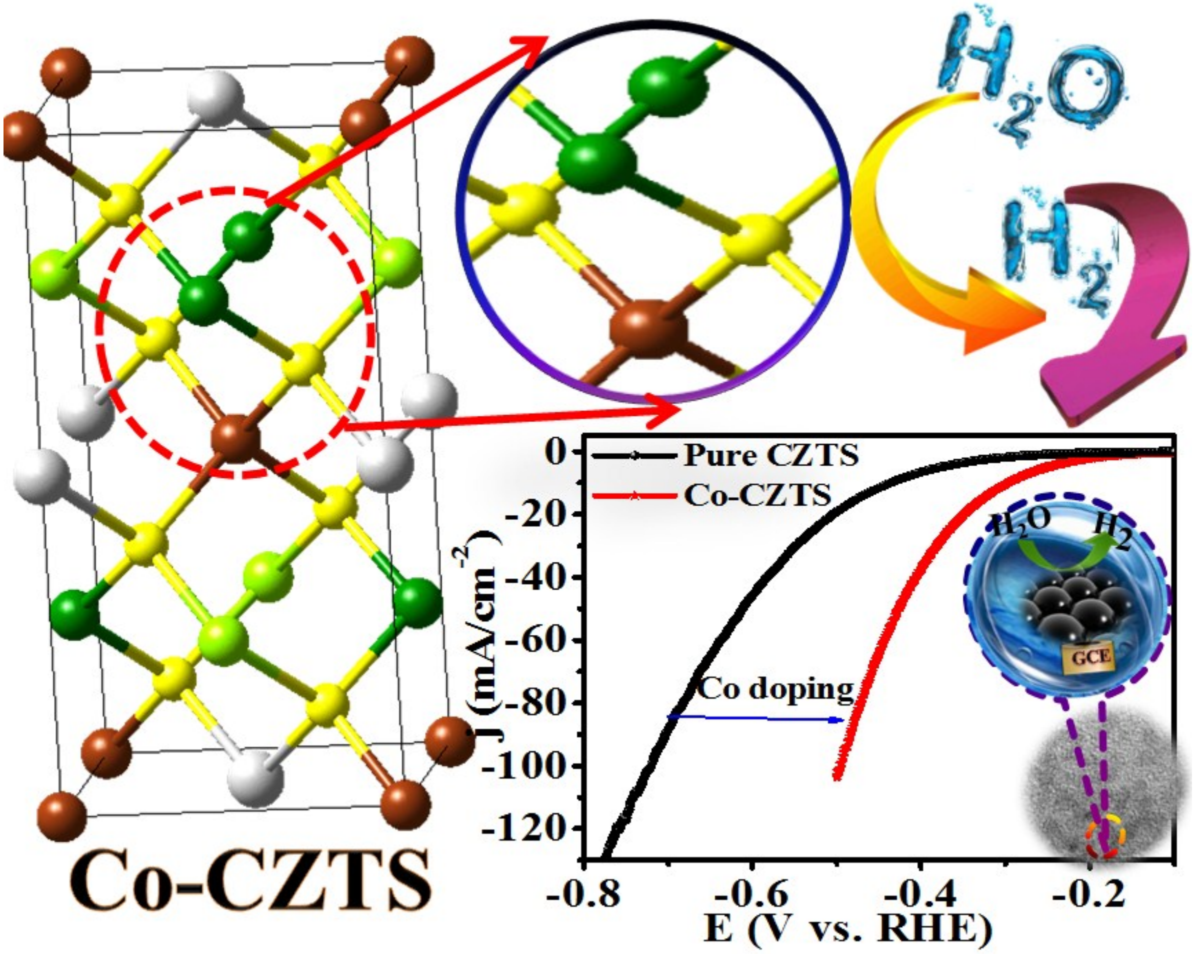

Scheme S1. Schematic representation of Co-doped CZTS for hydrogen evolution reaction.

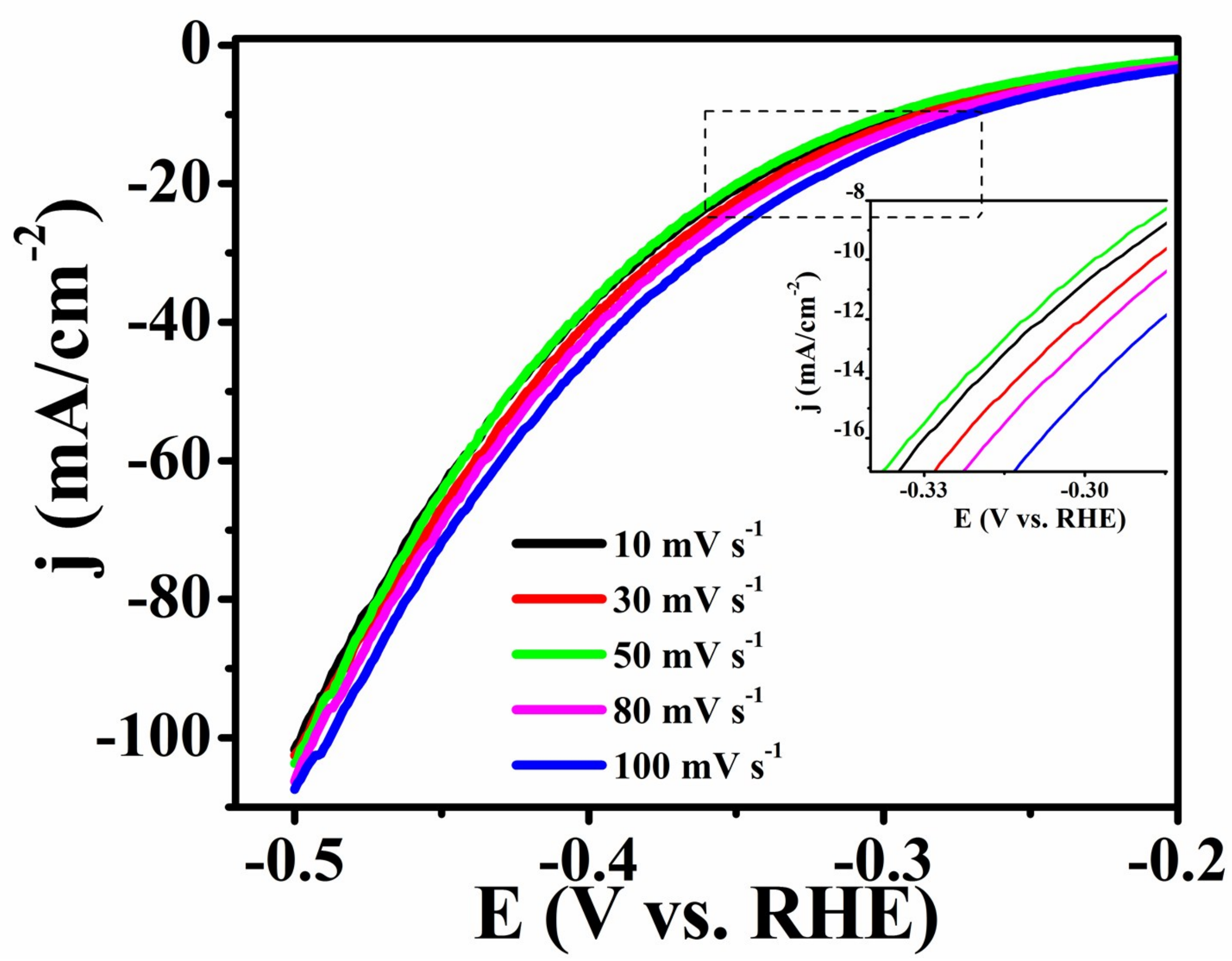

Fig. S2 Superimposed LSV of Co-doped CZTS nanoparticles with different scan rates i.e 10 to 100 mV s^{-1}

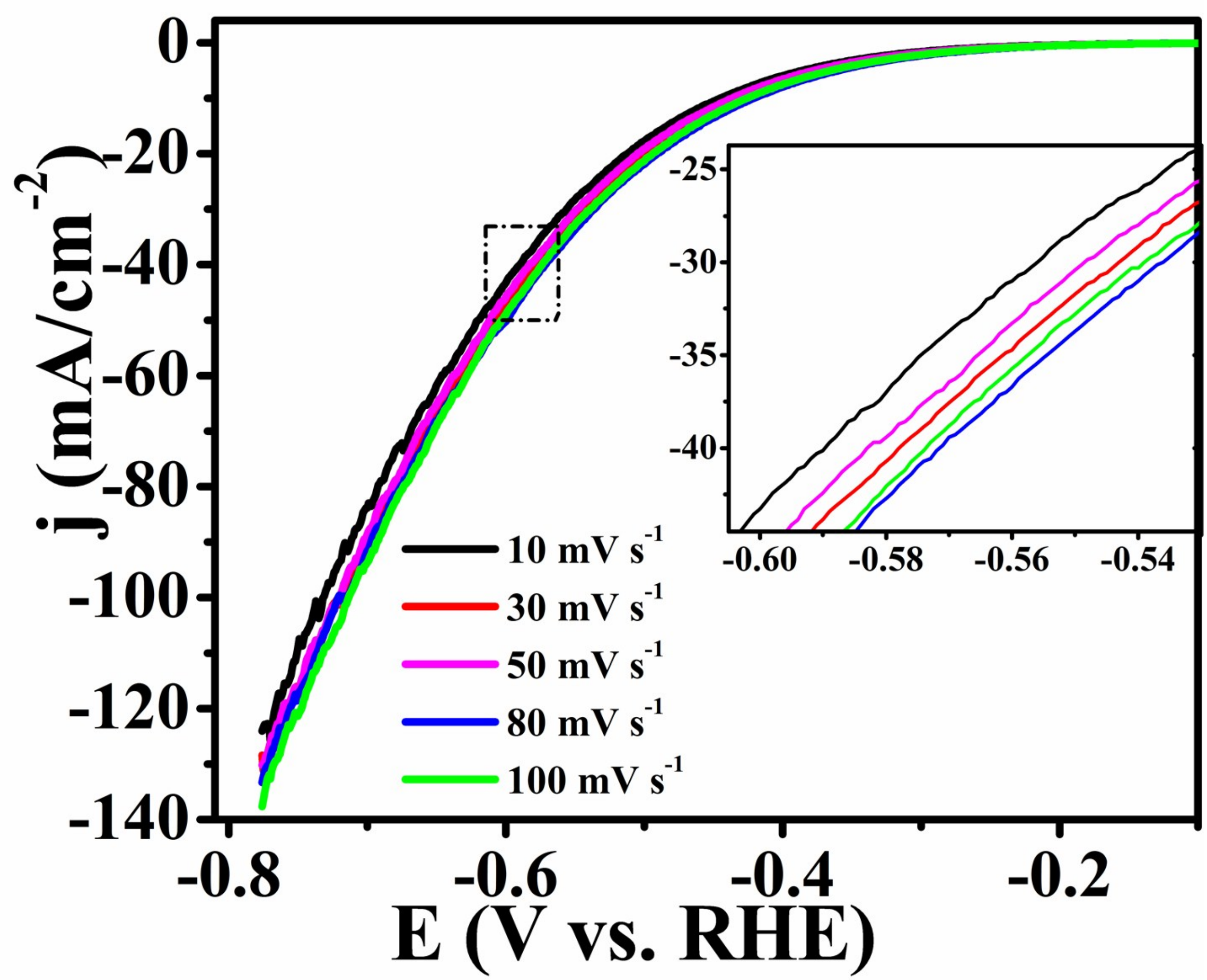

Fig. S3 Superimposed LSV of as synthesized CZTS nanoparticles at various scan rates i.e 10 to 100 mV s^{-1}.

Turn over frequency (TOF): Calculation of TOF is carried out based on an earlier reported. [5-7]

Turn over frequency (TOF)

Molar Mass=439.47 g.mol^{-1}

Density = 4.56g.cm^{-3}

Volume=96.37 ml.mol^{-1}

Current density at -0.3 V (300 mV overpotantial) = 0.188 x10^{-3}

Catalyst loading =5.71 mg.cm^{-2}

BET surface area 42.13 cm^{2}.mg^{-2}

Average surface atoms per 1 square centimeter

$$\left(\frac{3 \times 6.023 \times 10^{23}}{96.37}\right)^{2/3} atom.cm^{-2} = 7.05 \times 10^{14} atom.cm^{-2}$$

Surface atoms per tested area

$$5.71\ mg.cm^{-2} \times 42.13.mg^{-1} \times 7.05 \times 10^{14} atom.cm^{-2} = 0.169596 \times 10^{18} atom.cm^{-2}$$

Turnover Frequency (TOF)

$$\frac{1\ turnover\ \times 0.188 \times 10^{-3} A.cm^{-2} \times 1mol \times 6.023 \times 10^{23} e^{-}.mol^{-1}}{2e^{-}\ \times 96485C \times 0.169596 \times 10^{18} atom.cm^{-2}}$$

$$= \frac{1.132324 \times 10^{20}}{32726.94012 \times 10^{18}} = 0.188\ s^{-1}.atom^{-1}$$

Table S1. Comparison of literature turnover frequency (TOF) of various electrocatalysts.

Sr. No	Catalyst	TOF (s^{-1})	η (mV)	Ref.
1	MoS_2-160	0.012	220	[8]
2	Co-S	0.017	250	[9]
3	Cu_2ZnSnS_4 (CZTS)	0.0037	900	[7]
4	Nanoparticulate MoS_2	0.02	N/A	[10]
5	Co_3ZnC/RGO	0.0081	200	[11]
6	CoP	0.060	100	[12]
7	MoS_2-W3	0.0143	200	[13]
8	MoS_2 NSs-550	0.08	N/A	[14]
9	MoP	0.048	100	[15]
11	CZTS NPs	0.0703	300	[16]
12	Co-FL-MoS_2/NG	0.091	N/A	[17]
13	Co-B	0.088	250	[5]
14	MoS-C	0.13	200	[18]
15	MoS_2 nanosheets	0.198	200	[19]
16	MoSx/N-CFP	0.15	200	[20]
17	CoWS	0.12	300	[21]
18	Co-CZTS	0.188	300	**This Work**

References

1. W. Xie, X. Jiang, C. W. Zou, D. Li, J. Zhang, J. Quan and L. X. Shao, *Physica E*, 2012, **45**, 16-20.
2. M. Cao and Y. Shen, *J Cryst Growth*, 2011, **318**, 1117-1120.
3. M. J. Thompson, K. J. Blakeney, S. D. Cady, M. D. Reichert, J. D. Pilar-Albaladejo, S. T. White and J. Vela, *Chemistry of Materials*, 2016, **28**, 1668-1677.
4. C. Xiao, K. Li, J. Zhang, W. Tong, Y. Liu, Z. Li, P. Huang, B. Pan, H. Su and Y. Xie, *Materials Horizons*, 2014, **1**, 81-86.
5. S. Gupta, N. Patel, A. Miotello and D. Kothari, *J Power Sources*, 2015, **279**, 620-625.
6. E. J. Popczun, C. G. Read, C. W. Roske, N. S. Lewis and R. E. Schaak, *Angewandte Chemie*, 2014, **126**, 5531-5534.
7. P. Kush, K. Deori, A. Kumar and S. Deka, *J Mater Chem A*, 2015, **3**, 8098-8106.
8. D. Z. Wang, Z. Pan, Z. Z. Wu, Z. P. Wang and Z. H. Liu, *Journal of Power Sources*, 2014, **264**, 229-234.
9. Y. Sun, C. Liu, D. C. Grauer, J. Yano, J. R. Long, P. Yang and C. J. Chang, *Journal of the American Chemical Society*, 2013, **135**, 17699-17702.
10. T. F. Jaramillo, K. P. Jørgensen, J. Bonde, J. H. Nielsen, S. Horch and I. Chorkendorff, *science*, 2007, **317**, 100-102.
11. L. Ma, X. Shen, J. Zhu, G. Zhu and Z. Ji, *Journal of Materials Chemistry A*, 2015, **3**, 11066-11073.
12. E. J. Popczun, C. W. Roske, C. G. Read, J. C. Crompton, J. M. McEnaney, J. F. Callejas, N. S. Lewis and R. E. Schaak, *Journal of Materials Chemistry A*, 2015, **3**, 5420-5425.
13. J. N. He, Y. Q. Liang, J. Mao, X. M. Zhang, X. J. Yang, Z. D. Cui, S. L. Zhu, Z. Y. Li and B. B. Li, *J Electrochem Soc*, 2016, **163**, H299-H304.
14. Z. Z. Wu, B. Z. Fang, Z. P. Wang, C. L. Wang, Z. H. Liu, F. Y. Liu, W. Wang, A. Alfantazi, D. Z. Wang and D. P. Wilkinson, *Acs Catal*, 2013, **3**, 2101-2107.
15. X. B. Chen, D. Z. Wang, Z. P. Wang, P. Zhou, Z. Z. Wu and F. Jiang, *Chem Commun*, 2014, **50**, 11683-11685.

16. R. V. Digraskar, B. B. Mulik, P. S. Walke, A. V. Ghule and B. R. Sathe, *Appl Surf Sci*, 2017, **412**, 475-481.
17. L. Ma, L. Xu, X. Xu, X. Zhou, J. Luo and L. Zhang, *Materials Science and Engineering: B*, 2016, **212**, 30-38.
18. X. P. Dai, K. L. Du, Z. Z. Li, M. Z. Liu, Y. D. Ma, H. Sun, X. Zhang and Y. Yang, *Acs Appl Mater Inter*, 2015, **7**, 27242-27253.
19. X. Dai, K. Du, Z. Li, H. Sun, Y. Yang, W. Zhang and X. Zhang, *International Journal of Hydrogen Energy*, 2015, **40**, 8877-8888.
20. R. Bose, S. K. Balasingam, S. Shin, Z. Jin, D. H. Kwon, Y. Jun and Y.-S. Min, *Langmuir*, 2015, **31**, 5220-5227.
21. L. Yang, X. Wu, X. Zhu, C. He, M. Meng, Z. Gan and P. K. Chu, *Appl Surf Sci*, 2015, **341**, 149-156.
